# Prevalence of Dokha use in Arabian countries: A systematic review and meta-analysis

**DOI:** 10.1097/MD.0000000000051453

**Published:** 2026-11-06

**Authors:** Nazila Soufi Amlashi, Esrea H. Mahdy, Radhi K. Al-Rashidi, K.D.V. Prasad, Sunil G. Joshi, Vimal Arora, Amrita Pal, Sandeep Kumar Shukla, Samir Sahoo, Amirali Ebrahimi

**Affiliations:** a Guilan University of Medical Sciences, Rasht, Iran; b College of Pharmacy, The Islamic University, Najaf, Iraq; c Department of Laboratory Sciences, College of Dentistry, University of Kut, Wasit, Iraq; d Symbiosis Institute of Business Management, Hyderabad, India; e Symbiosis International (Deemed University), Pune, India; f Faculty of Ayurveda, Gokul Global University, Siddhpur, Gujarat, India; g University Institute of Pharma Sciences, Chandigarh University, Mohali, Punjab, India; h Department of Chemistry, Sathyabama Institute of Science and Technology, Chennai, Tamil Nadu, India; i Sharda School of Pharmacy, Sharda University, Greater Noida, India; j Department of General Medicine, IMS and SUM Hospital, Siksha “O” Anusandhan (Deemed to be University), Bhubaneswar, Odisha, India.

**Keywords:** Dokha, medwakh, midwakh, tobacco smoking, tobacco use

## Abstract

**Background:**

Dokha, a form of tobacco smoked using midwakh pipes, is increasingly used in Arabian countries, yet comprehensive prevalence data remain limited. This study aimed to systematically review and meta-analyze the prevalence of Dokha use across Arabian countries, stratified by gender, type of use, and use of specific smoking tools.

**Methods:**

A systematic literature search was conducted in PubMed, Scopus, Web of Science, and Embase for studies published in English from inception to June 2025. Eligible studies reported prevalence estimates of Dokha use among any population subgroup in Arabian countries. Two independent reviewers screened studies, extracted data, and assessed methodological quality using the Joanna Briggs Institute checklist. Random-effects meta-analyses were performed to estimate pooled prevalence rates for current and lifetime Dokha use among males and females, as well as use of specific Dokha smoking tools. Data were analyzed using Comprehensive Meta-Analysis version 3.0.

**Results:**

Out of 729 studies found, 12 studies were included, encompassing populations from multiple Arabian countries. Current Dokha use among females was extremely low (0.7%, 95% confidence interval [CI] = 0.1%–4.9%), whereas males exhibited substantially higher current use (14.6%, 95% CI = 7.9%–25.4%). Lifetime use followed similar trends, with a pooled prevalence of 9.1% (95% CI = 3.9%–20.0%) among females and 25.6% (95% CI = 10.8%–49.5%) among males. Use of specific Dokha smoking tools was reported at 7.3% (95% CI = 5.6%–9.4%) for current use and 15.8% (95% CI = 10.0%–24.0%) for lifetime use. Considerable heterogeneity was observed across studies, particularly among male users, reflecting differences in population, geography, and study methodology.

**Conclusion:**

Dokha use in Arabian countries demonstrates pronounced gender disparities, with males showing substantially higher prevalence than females, which highlights the need for targeted public health interventions, surveillance, and culturally sensitive tobacco control strategies addressing Dokha use in this region.

## 1. Introduction

Tobacco use remains a leading cause of preventable morbidity and mortality worldwide, contributing to cardiovascular disease, cancer, respiratory disorders, and numerous other health complications.^[1,2]^ While cigarette smoking has received considerable attention, alternative forms of tobacco, including waterpipe and Dokha (also known as midwakh/medwakh), have emerged as culturally specific yet increasingly prevalent modes of consumption, particularly in the Arabian Peninsula.^[3,4]^ Dokha is a finely shredded blend of tobacco, herbs, and spices that is smoked through a small pipe known as a midwakh. Its unique composition and high nicotine content make it potentially more addictive and harmful than conventional cigarettes, yet public awareness and research on its use remain limited.^[5,6]^

The use of Dokha has deep cultural roots in the Arabian region, especially in countries such as the United Arab Emirates, Saudi Arabia, Oman, Qatar, and Kuwait.^[6,7]^ Studies suggest substantial variability in prevalence, reflecting differences in population demographics, age, gender, urbanization, and cultural norms. Surveys among adolescents and young adults in Arabian countries have reported prevalence rates of current tobacco use ranging from 3% to nearly 41% among males, whereas female usage remains consistently low. Lifetime use estimates similarly indicate higher exposure among males compared to females.^[4,8,9]^ Despite these observations, comprehensive and pooled prevalence data across Arabian countries are lacking, limiting evidence-based public health interventions.

Dokha use is influenced by a combination of biological, behavioral, and sociocultural factors. Nicotine dependence, peer influence, accessibility of Dokha, and social acceptability in specific male-dominated contexts contribute to the initiation and continuation of use.^[10,11]^ In addition, exposure to advertising, lack of regulation, and limited awareness of health risks exacerbate consumption patterns. Smoking Dokha has been associated with acute cardiovascular effects, respiratory symptoms, and elevated biomarkers of nicotine exposure, although long-term population-level health impacts remain under-investigated.^[12,13]^

Given the increasing popularity of Dokha and the potential public health implications, it is essential to synthesize existing evidence on its prevalence, patterns, and determinants. This systematic review and meta-analysis aimed to provide a comprehensive evaluation of Dokha use across Arabian countries.

## 2. Materials and methods

### 2.1. Study design

This study was conducted as a systematic review and meta-analysis to estimate the prevalence of Dokha use in Arabian countries. The study adhered to the Preferred Reporting Items for Systematic Reviews and Meta-Analyses guidelines to ensure methodological rigor and transparency.^[14]^

### 2.2. Literature search strategy

A comprehensive literature search was performed in multiple electronic databases, including PubMed, Scopus, Web of Science, and Embase. The literature search was conducted using a combination of keywords and Medical Subject Headings (MeSH) terms to capture studies on Dokha use. The search terms included “Dokha,” “Midwakh,” “Medwakh,” “Midwakh smoking,” “Tobacco, Smokeless” [MeSH], “Tobacco Use” [MeSH], “Tobacco Smoking” [MeSH], and “Tobacco Use Disorder” [MeSH], combined with terms related to prevalence and study design such as “Prevalence,” “Epidemiology,” “Cross-Sectional Studies” [MeSH], “Survey,” “Population,” “Frequency,” “Rate,” and “Patterns.” To ensure comprehensive coverage of the geographical focus, terms representing Arab countries were included: “Arabia,” “Arab countries,” “Middle East,” “Gulf States,” “GCC Countries,” “United Arab Emirates,” “Saudi Arabia,” “Qatar,” “Oman,” “Kuwait,” “Bahrain,” “Jordan,” “Lebanon,” “Iraq,” “Yemen,” “Syria,” “Palestine,” “West Bank,” “Gaza,” “Egypt,” “Sudan,” “Libya,” “Tunisia,” “Algeria,” “Morocco,” “Mauritania,” “Djibouti,” “Somalia,” and “Comoros.” Searches were limited to studies conducted in Arabian countries and published in English from inception to June 2025. Additional studies were identified by screening reference lists of relevant articles.

### 2.3. Eligibility criteria

Studies were included if they reported prevalence estimates of Dokha use among any population subgroup in Arabian countries, employed cross-sectional, cohort, or survey-based designs, provided sufficient data to extract prevalence rates for current and/or lifetime use, and clearly defined the study population and sampling method. Studies were excluded if they were case reports, case series, editorials, or reviews, conducted outside Arabian countries, provided insufficient or unclear data on Dokha prevalence, or were duplicate publications.

### 2.4. Study selection and data extraction

Two independent reviewers screened titles and abstracts for eligibility, followed by full-text review (κ = 0.72), indicating substantial inter-reviewer agreement. Discrepancies were resolved through discussion or consultation with a third reviewer. From eligible studies, the following data were extracted: author, year of publication, country, study population, sample size, age range, gender distribution, prevalence of current and lifetime Dokha use, and prevalence of use of specific Dokha smoking tools. Data were stratified by gender and type of use to allow subgroup analyses.

### 2.5. Quality assessment

The methodological quality of included studies was assessed using a modified version of the Joanna Briggs Institute Critical Appraisal Checklist for Prevalence Studies.^[15]^ The assessment considered sampling method, sample representativeness, measurement reliability, and statistical reporting. Studies were classified as high, moderate, or low quality based on the total score, and sensitivity analyses were conducted to evaluate the influence of study quality on pooled prevalence estimates.

### 2.6. Statistical analysis

Results were reported as pooled prevalence rates with 95% confidence intervals (CIs). Forest plots were generated to illustrate the prevalence estimates and associated heterogeneity for each subgroup. Meta-analyses were performed using a random-effects model to account for anticipated heterogeneity across studies. Prevalence estimates were pooled separately for males and females, for current and lifetime use, and for the use of specific Dokha smoking tools. Heterogeneity was quantified using the *I*^2^ statistic and Cochran’s *Q* test. Subgroup analyses and meta-regression were conducted to explore potential sources of heterogeneity, including geographic region, age group, and study period. Publication bias was assessed visually using funnel plots and quantitatively using Egger’s test. All analyses were performed using Comprehensive Meta-Analysis version 3.0.

## 3. Results

The systematic review identified 729 studies, of which 12 were included for meta-analysis that examined the prevalence of Dokha across Arabian countries (Fig. 1). The analysis included multiple studies with data stratified by gender and usage patterns (current use vs ever use). Quality assessment using the Joanna Briggs Institute checklist^[16]^ illustrated that the majority of studies demonstrated high methodological quality, although some items were rated as No or Unclear, indicating potential risks of bias in specific domains (e.g., sample representativeness or reporting clarity).

**Figure 1.**
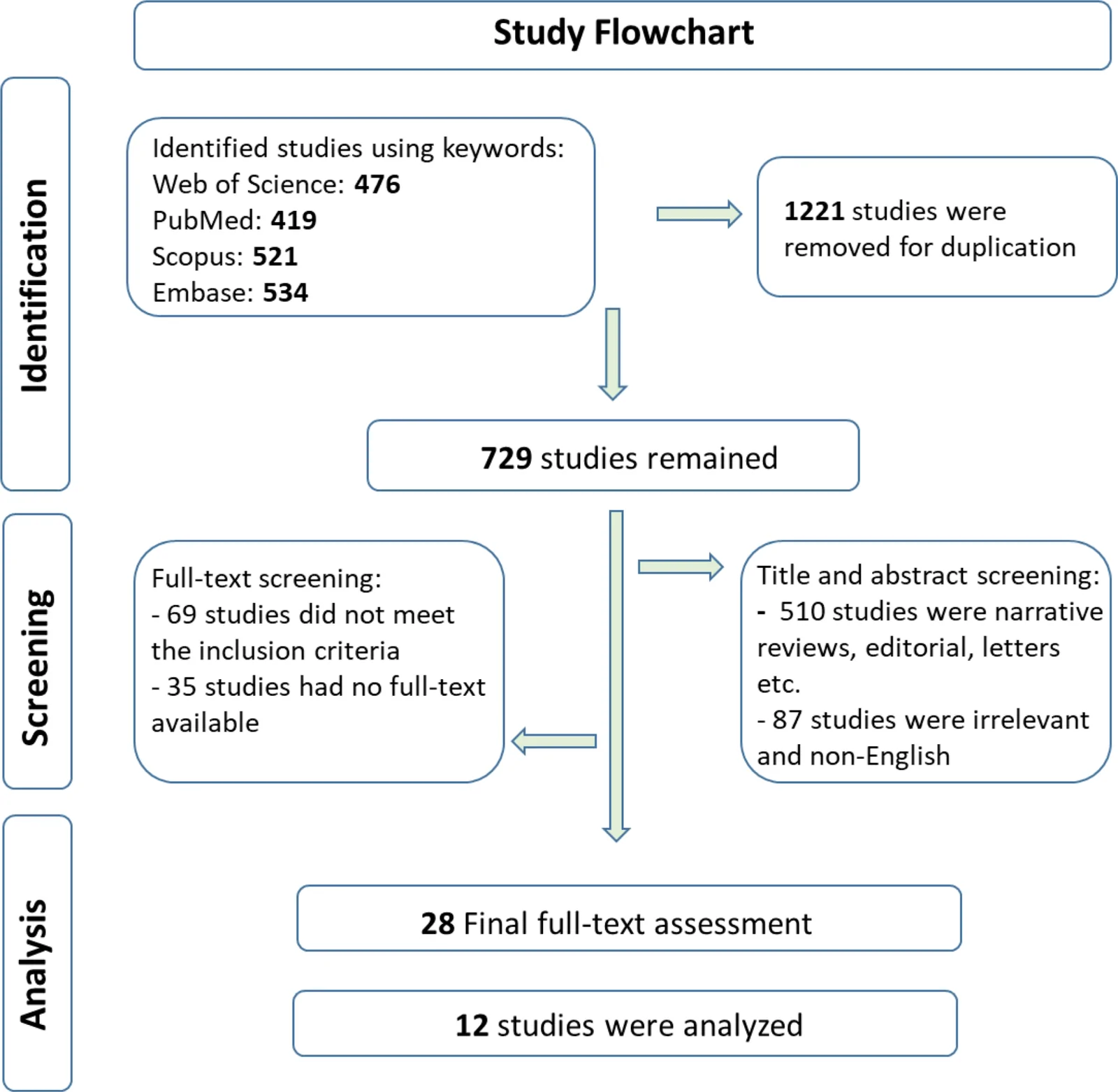
Flowchart of literature selection process.

### 3.1. Quality assessment

Overall, the methodological quality of the included studies was high. Ten of the 12 studies were classified as high quality. Most studies adequately addressed the target population, sampling methods, sample size, data analysis, and the validity and reliability of outcome measurement. However, a few studies showed potential methodological limitations related to sample representativeness and insufficient reporting of certain methodological domains, resulting in some items being rated as “No” or “Unclear” (Fig. 2).

**Figure 2.**
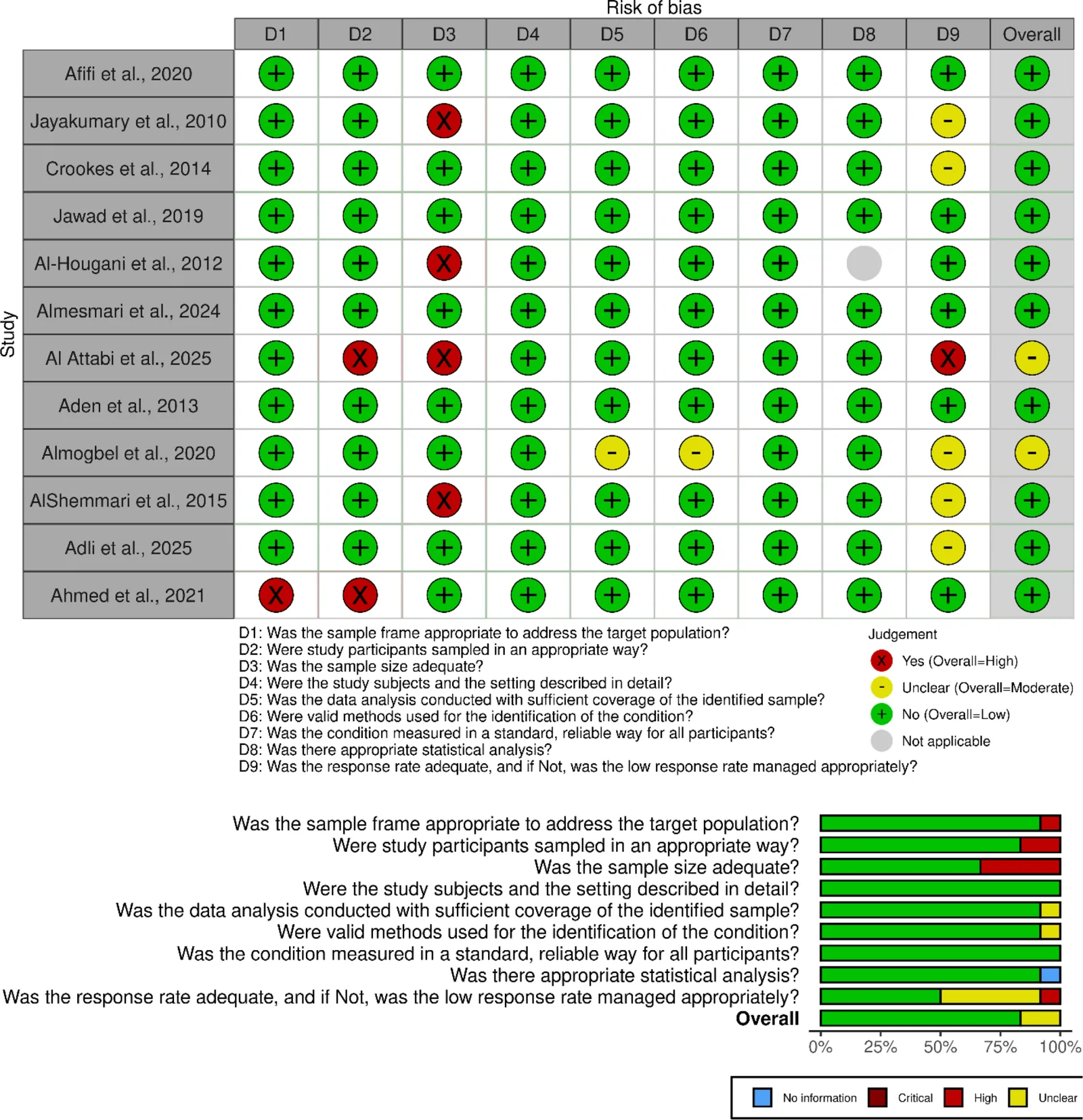
The results of the risk of bias assessment.

### 3.2. Prevalence of current Dokha use among females

Data on study characteristics have been reported in Table 1. The meta-analysis of current Dokha use among females included 4 studies.^[7,17,21,22]^ The pooled prevalence estimate revealed extremely low current use among females, with a meta-analytic rate of 0.007 (95% CI = 0.001–0.049), indicating that <1% of females in the studied populations were current Dokha users. Single study estimates ranged from 0.001 in both Al-Houqani et al^[17]^ and Aden et al^[22]^ studies to 0.037 (95% CI = 0.031–0.044) in the study by Afifi et al^[21]^ (Fig. 3A).

**Table 1:** Studies characteristics.

Study	Location	Year	Sample size	Age group	Study type	Gender	Prevalence/key findings
Al-Houqani et al^[17]^	UAE (Abu Dhabi)	2009–2010	170,430	≥18 years	Cross-sectional	Both	15% midwakh use among smokers; 1.7% daily midwakh users
Jayakumary et al^[18]^	UAE (Ajman)	2009	104	University students	Cross-sectional	Both	11.5% lifetime Dokha use; 30.4% male vs 5.1% female
Crookes and Wolff^[19]^	UAE (Dubai)	2010–2012	394	High school (15–19)	Cross-sectional	Both	54.8% of tobacco users smoked Dokha
Al Shemmari et al^[20]^	UAE (Ajman)	Not specified	560	Secondary school	Cross-sectional	Male only	39% ever smokers; 36% ever Dokha; 24% current Dokha
Jawad et al^[7]^	UAE	2013	4259	13–15 years	Cross-sectional	Both	18.5% ever use; 9.0% current use
Afifi et al^[21]^	Lebanon	2017	5590	Grades 7–12 (12–18)	Cross-sectional	Both	4.6% current midwakh use
Aden et al^[22]^	UAE (Abu Dhabi)	2011	9337	Adults	Cross-sectional	Both	5.9% midwakh smoking
Ahmed et al^[8]^	UAE	2019–2020	1123	University students	Cross-sectional	Both	15.1% current smokers, 4.0% current nicotine vaping, 28.8% poly-users, 47.5% conventional midwakh, 36.7% conventional hisha/waterpipe, 36.7% conventional cigarettes, 25.2% electronic shisha/waterpipe, 24.5% electronic cigarettes
Almogbel et al^[4]^	Saudi Arabia (Qassim)	2018	316	University male students	Cross-sectional	Male	7.9% ever smokers; 3.8% current smokers
AL Attabi et al^[23]^	Oman (Sultan Qaboos University)	2013–2020	63	College students	Cross-sectional	Mostly male	98.4% male; median age 16 at initiation
Almesmari et al^[24]^	UAE (Military)	2021–2022	369	Military recruits (17–23)	Cross-sectional	Male	41.6% smoking prevalence
Adli et al^[25]^	Qatar	2020–2021	118/576	≥18 years	Cross-sectional	Both	4.4% midwakh users among current smokers

UAE = United Arab Emirates.

**Figure 3.**
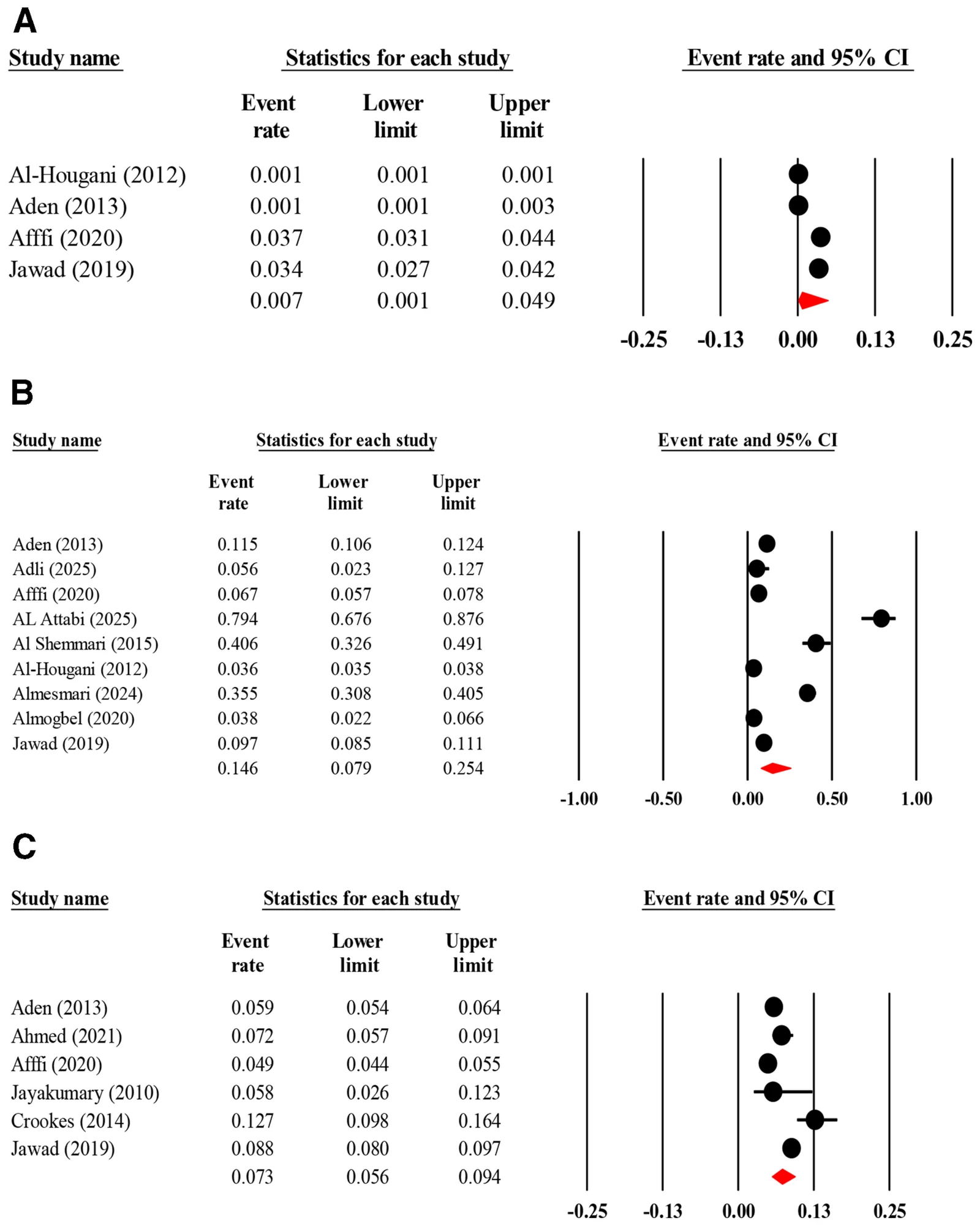
Prevalence of current Dokha use in Arabian countries. (A) Females. (B) Males. (C) Specific Dokha smoking tools. CI = confidence interval.

### 3.3. Prevalence of current Dokha use among males

Current Dokha use among males demonstrated substantially higher prevalence rates compared to females. The meta-analysis incorporated 9 studies and yielded a pooled prevalence of 14.6% (95% CI = 7.9%–25.4%).^[4,7,17,20–25]^ Notable heterogeneity was observed across studies, with individual study prevalence rates ranging from 3.6% (95% CI = 3.5%–3.8%)^[17]^ to as high as 79.4% (95% CI = 67.6%–87.6%).^[23]^ Other significant findings included the study by Al Shemmari et al^[20]^ with 40.6% prevalence (95% CI = 32.6%–49.1%) and Almesmari et al^[24]^ with 35.5% prevalence (95% CI = 30.8%–40.5%; Fig. 3B).

### 3.4. Prevalence of current use of specific Dokha smoking tools

The analysis of current use of specific Dokha smoking tools included 6 studies,^[7,8,18,19,21,22]^ which demonstrated a pooled meta-analytic estimate of 7.3% (95% CI = 5.6%–9.4%) using specific Dokha smoking tools. Individual study estimates were relatively consistent, ranging from 4.9% (95% CI = 4.4%–5.5%)^[21]^ to 12.7% (95% CI = 9.8%–16.4%).^[19]^ The consistency across studies suggests a stable pattern of lifetime experimental or occasional use of specific Dokha smoking tools in Arabian countries (Fig. 3C).

### 3.5. Prevalence of lifetime Dokha use among females

Lifetime Dokha use among females was examined in 2 studies,^[7,18]^ revealing a pooled prevalence of 9.1% (95% CI = 3.9%–20.0%). The wide CI suggests considerable heterogeneity between the included studies. Jawad et al^[7]^ reported 12.5% lifetime prevalence (95% CI = 11.2%–13.9%), while Jayakumary et al^[18]^ reported 5.1% (95% CI = 1.9%–12.9%; Fig. 4A).

**Figure 4.**
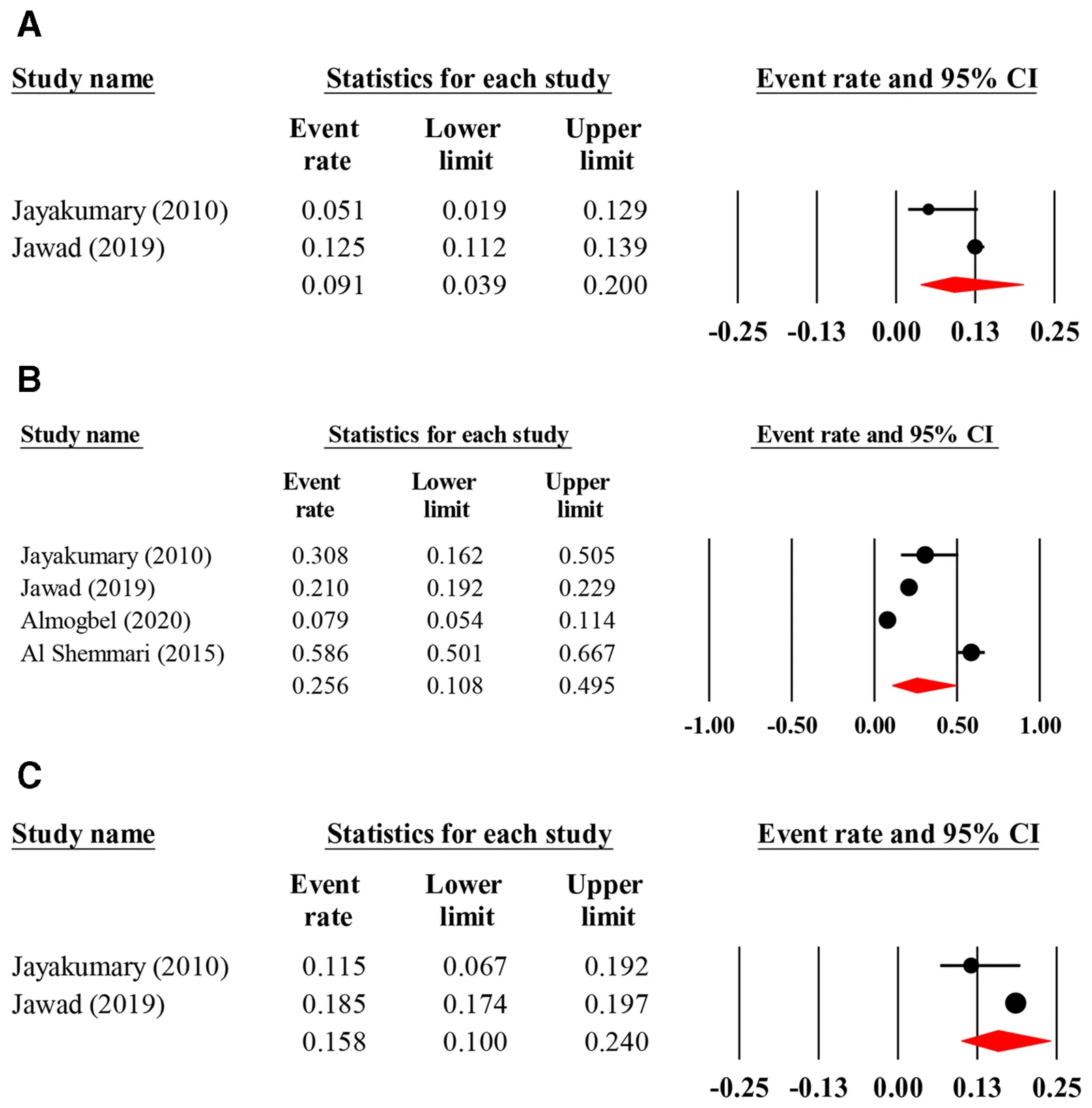
Prevalence of lifetime Dokha use in Arabian countries. (A) Females. (B) Males. (C) Specific Dokha smoking tools. CI = confidence interval.

### 3.6. Prevalence of lifetime Dokha use among males

The analysis examining the prevalence of Dokha use among males included 4 studies and demonstrated a pooled prevalence of 25.6% (95% CI = 10.8%–49.5%).^[4,7,18,20]^ This category showed the highest variability, with Al Shemmari et al^[20]^ reporting 58.6% prevalence (95% CI = 50.1%–66.7%), and Almogbel et al^[4]^ reporting only 7.9% (95% CI = 5.4%–11.4%; Fig. 4B).

### 3.7. Prevalence of lifetime use of specific Dokha smoking tools

The analysis of lifetime use of specific Dokha smoking tools included 2 studies^[7,18]^ and yielded a pooled estimate of 15.8% (95% CI = 10.0%–24.0%). The study by Jawad et al^[7]^ contributed the larger estimate at 18.5% (95% CI = 17.4%–19.7%), while Jayakumary et al^[18]^ reported 11.5% (95% CI = 6.7%–19.2%; Fig. 4C).

### 3.8. Publication bias

The funnel plot (Fig. 5) demonstrated noticeable asymmetry, suggesting the possibility of small-study effects or publication bias. This observation was supported by Egger’s regression test, which showed statistically significant evidence of publication bias (*P* = .033, two-tailed). Therefore, the pooled prevalence estimates should be interpreted with caution, as selective publication of studies with higher or more significant prevalence estimates may have influenced the overall results. In addition, the substantial between-study heterogeneity observed in several subgroup analyses may have contributed to the funnel plot asymmetry and should also be considered when interpreting the findings.

**Figure 5.**
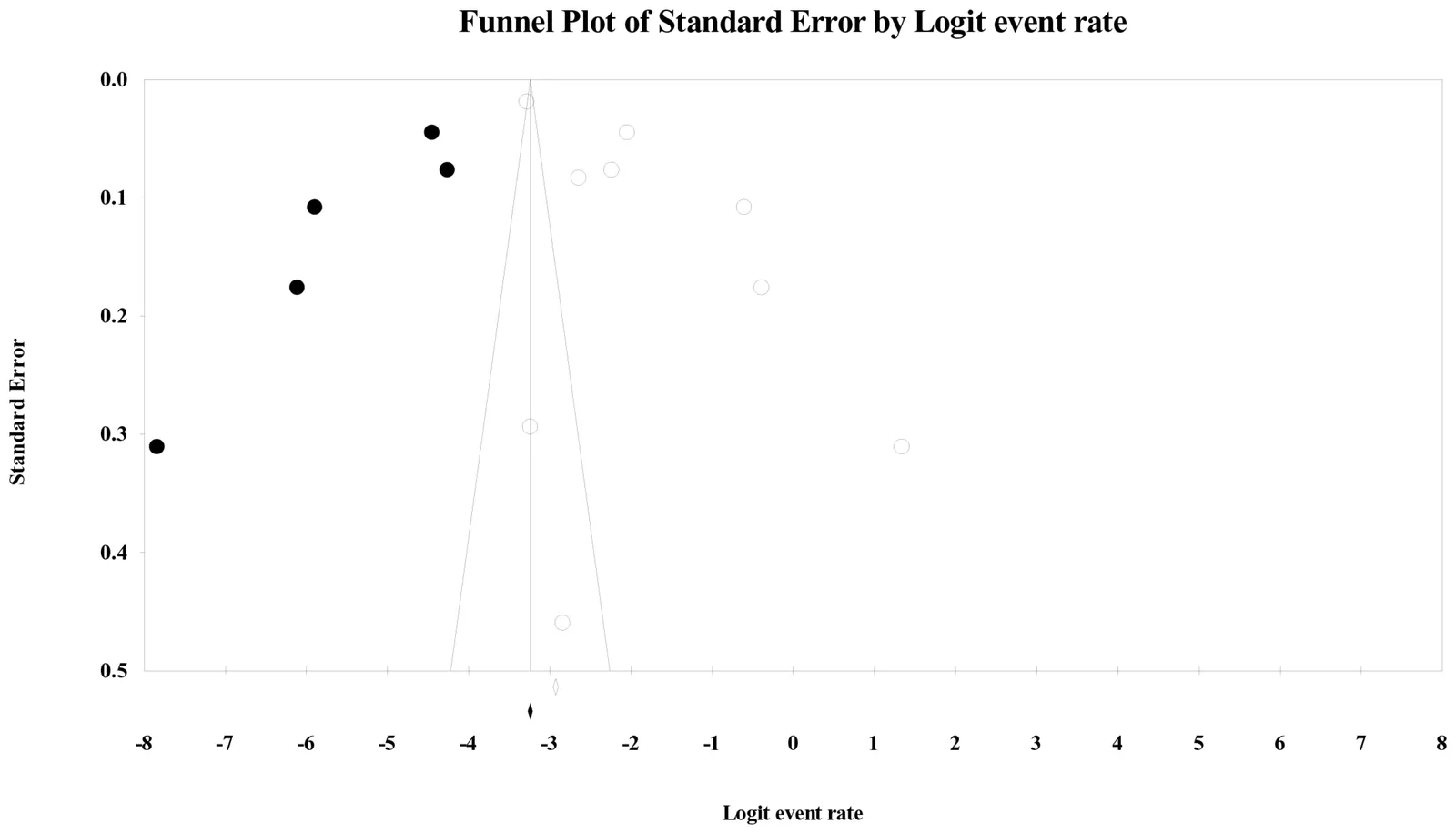
Funnel plot of observed studies on the prevalence of Dokha use.

## 4. Discussion

This systematic review and meta-analysis is the first to analyze data on the prevalence of Dokha use across Arabian countries, providing gender-stratified estimates and highlighting usage patterns. The findings reveal a striking gender disparity, with current Dokha use among females being rare, while males exhibit substantially higher prevalence for lifetime use. The results demonstrate a pronounced gender disparity, with current male use being approximately 20-fold higher than current female use. This gap was still evident in lifetime use patterns, underscoring the strong gendered dynamics in tobacco use behaviors. These findings are consistent with broader regional tobacco use surveys that document significantly higher smoking prevalence among men compared to women in the Middle East.^[26,27]^

The results from national tobacco surveys conducted in Gulf Cooperation Council countries illustrated that tobacco use, regardless of product type, is lower among females compared to males.^[28,29]^ Cultural, social, and religious norms may play a pivotal role in constraining female smoking behaviors, a factor repeatedly emphasized in previous reviews on tobacco use in the region.^[30,31]^ This observation is reinforced by the consistently narrow CIs in female prevalence estimates, which reflect low variability across studies and indicate that female tobacco use is not only rare but also socially constrained in most Arabian contexts.

Compared with waterpipe and cigarette smoking, the observed prevalence of Dokha use among males appears remarkably high. A systematic review by Jawad et al^[7]^ on waterpipe smoking reported a lower prevalence of Dokha use among male adults in Middle Eastern countries compared to our findings. We also found a high prevalence of Dokha use compared to the report from the 2021 Saudi National Health Survey on currently smoked tobacco and smokeless tobacco use.^[32]^ This suggests that Dokha may not only be a culturally entrenched practice but also a potentially underrecognized driver of the tobacco epidemic in Arabian countries, with health risks likely amplified by the higher frequency and intensity of use compared to conventional tobacco products.^[33,34]^

In the current study, the heterogeneity observed in male prevalence underscores the influence of contextual and methodological factors. High-prevalence studies, such as Al Shemmari et al,^[20]^ likely reflect subpopulations with greater exposure to Dokha, such as university students or younger adults, echoing patterns reported in other systematic reviews on waterpipe smoking that noted prevalence peaks among youth and college students.^[35,36]^ By contrast, lower prevalence estimates by Al-Houqani et al^[17]^ indicate that Dokha use may not be uniformly widespread across all demographic groups. This heterogeneity is also evident in the wide CIs of pooled estimates for male current use, suggesting substantial between-study variability that may be influenced by geographic region, population characteristics, and temporal shifts in tobacco consumption over the 2010 to 2025 study period.^[37,38]^

Regarding lifetime Dokha use, our pooled prevalence estimates demonstrate that experimentation or intermittent use is more common than current use, particularly among women. This trajectory mirrors that of waterpipe smoking, where lifetime prevalence far exceeds current use due to experimentation during adolescence or early adulthood, followed by later cessation or transition to other tobacco products.^[35,39]^ The higher lifetime prevalence also highlights the need to distinguish between experimental versus habitual use, as has been stressed in systematic reviews of youth tobacco behaviors in the region.^[40,41]^

In the current study, the prevalence of current tool use and lifetime tool use suggests that experimentation with smoking implements is relatively stable across studies, reflecting a cultural pattern of social smoking practices. The consistency of prevalence rates across studies indicates a relatively uniform pattern of use, in contrast to the wide variation observed in male current use. This stability may reflect the cultural normalization of Dokha paraphernalia such as the medwakh pipe, which reinforces identity and social cohesion in smoking rituals. This finding parallels systematic reviews of shisha and medwakh smoking, which note that the unique paraphernalia associated with these products contribute to their cultural identity and continued popularity.^[17,21]^

Studies on the health effects of alternative tobacco products, including Dokha, consistently show harmful cardiopulmonary and oral health outcomes, often exceeding those of cigarettes due to higher nicotine concentrations and combustion byproducts.^[3,42,43]^ The relatively high prevalence of Dokha use identified in our study therefore underscores an urgent need for targeted surveillance, regulation, and health education interventions in Arabian countries. The fact that waterpipe and cigarette smoking have received more consistent policy attention, while Dokha remains under-regulated, highlights a critical gap in public health response, particularly given its addictive potential and its popularity among young males.^[35,39]^

Our study emphasizes the gendered nature of tobacco use, the importance of considering lifetime versus current prevalence, and the cultural embeddedness of specific tobacco products. However, this study has several limitations that should be acknowledged. First, the number of eligible studies was limited, particularly for females, which may have reduced the precision of pooled estimates. Second, substantial heterogeneity was observed across included studies, likely due to differences in study populations, settings (e.g., students vs community samples), and measurement tools used to define Dokha use. Third, most studies relied on self-reported data, which are subject to recall and social desirability bias, especially among women in conservative cultural contexts. Finally, the lack of standardized definitions of current and lifetime Dokha use across studies may have contributed to variability in prevalence estimates.

The wide CIs observed for several pooled estimates further suggest that the precision of our results is limited and should be interpreted with caution. Future research should prioritize standardizing measurement of Dokha use, examining age- and occupation-specific subgroups, and assessing longitudinal trajectories of use. These steps will facilitate more accurate monitoring and support the development of targeted public health policies aimed at curbing the rising burden of alternative tobacco use in Arabian countries.

## 5. Conclusion

This systematic review and meta-analysis demonstrated that Dokha use is notably more prevalent among males than females across Arabian countries, with substantial variability between studies. Current use among females was extremely low, whereas nearly 1 in 7 males reported current use, and lifetime use reached 1 in 4. The findings highlight the cultural and gender-specific patterns of Dokha consumption in the region and underscore the need for targeted public health interventions and policies to address its rising use.

## Acknowledgments

DeepSeek AI was chiefly used for the initial polishing of language and correction of grammar, whereas Qwen AI 3.7 Plus was employed to further enhance sentence structure, academic tone, and overall readability. The prompts given to these tools were limited to editing text already written by the authors, with the goal of improving clarity and adherence to scientific writing conventions. None of the prompts were used to generate new content, data, or conclusions.

## Author contributions

**Conceptualization:** Nazila Soufi Amlashi, Amirali Ebrahimi.

**Investigation:** Radhi K. Al-Rashidi, Sunil G. Joshi, Vimal Arora.

**Methodology:** Radhi K. Al-Rashidi, Vimal Arora, Samir Sahoo.

**Software:** K.D.V. Prasad.

**Visualization:** K.D.V. Prasad.

**Formal analysis:** Sunil G. Joshi.

**Data curation:** Samir Sahoo.

**Supervision:** Amirali Ebrahimi.

**Writing –original draft:** Nazila Soufi Amlashi, Esrea H. Mahdy, Amrita Pal, Sandeep Kumar Shukla, Samir Sahoo.

**Writing – review & editing:** Nazila Soufi Amlashi, Esrea H. Mahdy, Amrita Pal, Sandeep Kumar Shukla.
